# Long-Term Results and Quality of Life after Surgery for Acute Aortic Dissection Type A: Contemporary Single-Centre Experience

**DOI:** 10.3390/jcm13185645

**Published:** 2024-09-23

**Authors:** Nora Goebel, Simone A. Holder, Franziska Huether, Eleanor Maw, Rafael Ayala, Yasemin Anguelov, Ulrich F. W. Franke, Dorothee Bail

**Affiliations:** 1 Department of Cardiovascular Surgery, Robert Bosch Hospital, 70376 Stuttgart, Germany; 2 Warwick Medical School, University of Warwick, Coventry CV4 7AL, UK; 3 Robert Bosch Society for Medical Research, Bosch Health Campus, 70376 Stuttgart, Germany; 4 Department of Cardiovascular Surgery, University Heart Center Freiburg Bad Krozingen, 79189 Bad Krozingen, Germany; ulrich.franke@uniklinik-freiburg.de

**Keywords:** type A aortic dissection, aortic surgery, long-term results, outcomes, reintervention, quality of life

## Abstract

**Background**: Aortic dissection is still afflicted with significant morbidity and mortality. This research seeks to assess long-term outcomes and quality of life after emergency surgery for acute aortic dissection type A. **Methods**: A total of 413 patients were analysed, who had been operated upon between 2000 and 2016 at our centre. We compared our results of the early (2000–2007) versus late (2008–2016) period with regards to 30-day and follow-up mortality and need for reoperation, including risk factor analysis. Quality of life was assessed via the SF-36 survey. **Results**: Calculated perioperative risk by EuroSCORE increased significantly from early, 24.9%, to late, 38.0%, *p* < 0.001. Thirty-day rates of mortality decreased significantly from 26.7% to 17.4%, *p* = 0.03. Survival at 1-, 5-, and 10-years was 92.3% vs. 91.8% (*p* = 0.91), 75.2% vs. 81.0% (*p* = 0.29), and 53.4% vs. 69.7% (*p* = 0.04). Freedom from reoperation was comparable between groups at follow-up: 74.0% vs. 85.7%, *p* = 0.28. Quality of life was impaired. **Conclusions**: Despite more complex severity of disease and operative procedures, the results of surgery for type A aortic dissection improved significantly over time at 30-day and 10-year follow-up. Quality of life was significantly impaired compared to a healthy reference population.

## 1. Introduction

Despite significant improvements in the understanding of the disease, diagnosis and therapy, the perioperative mortality and complication rates of type A aortic dissection surgery remain high, with 30-day mortality rates ranging between 15 and 30% [1]. Nevertheless, they are significantly below the mortality rates of the disease’s natural progression, which are as high as 55% within the first 48 h [2,3]. This is why surgery remains the therapy of choice and should not be dismissed lightly. However, some perioperative details, like timing, extent of surgical repair, temperature, or perfusion management, still present numerous uncertainties regarding optimal treatment [1]. Even so, the reported 30-day mortality rates after surgery seem to decrease slowly but steadily over time [4,5]. Beyond that, there are significant differences between low- and high-volume centres as well as between geographic regions [6,7,8,9,10,11]. The reasons for these disparities are multifactorial and not fully understood. Undoubtedly, surgical techniques have evolved significantly in the last two decades, with the frozen elephant trunk being the most prominent and controversially discussed player entering the stage [12,13]. However, its role and potential benefits in the long term have not been proven so far [14].

Moreover, aortic dissection patients experience significant long-term morbidity and mortality, which is very likely under-estimated [15,16]. The impact of aortic dissection and its major surgical intervention on quality of life in surviving patients has been described in small cohorts for the early postoperative course but is largely unknown in the long term and not comparable to other cardiovascular surgeries, where usually a rapid improvement in quality of life can be seen [17,18]. Owing to the emergent nature of the disease and this surgery, no RCT data exist. As a result, registries and high-volume single centre data can offer significant insights. We hypothesized that the evolution of surgical techniques had an impact on outcomes and therefore analysed our centre’s results with regards to early and late mortality, reintervention, and quality of life over a 17-year period.

## 2. Materials and Methods

### 2.1. Patients

A total of 413 patients had undergone surgery for acute type A aortic dissection at our centre, Robert Bosch Hospital, Stuttgart, Germany, between 2000 and 2016, and were included. These were allocated to early (2000–2007, *n* = 131) and late (2008–2016, *n* = 282) period groups, which were then compared. Timeframes were set as such for two reasons: (1) most of the modifications in operative management were introduced with a restructuring of the surgical program in 2007, and (2) to cover each for approximately the same amount of time in years, roughly 8 years before and after. All aortic patients undergo follow-up visits in our outpatient clinic on a regular, generally annual, basis.

### 2.2. Endpoints

We assessed early and late follow-up outcomes, including mortality, reintervention rate, and quality of life. Mortality rates were assessed for 30 days and in long-term follow-up at 1-, 5-, 10-, up to 12.5 years. Reintervention was defined as any operative or interventional surgery on the aorta during follow-up, irrespective of the location proximal or distal.

Risk factor analysis using multivariate regression was performed for early and late mortality. The following variables were included in the regression model: age, sex, BMI (body mass index), DeBakey type, preoperative CPR (cardiopulmonary resuscitation), shock, pericardial tamponade, malperfusion, neurologic deficit, histopathology, diabetes, COPD (chronic obstructive pulmonary disease), coronary artery disease, previous cardiac operation, operative time, crossclamp-time, circulatory arrest time, lowest temperature, cerebral perfusion, arterial cannulation site, postoperative mechanical circulatory support, aortic root surgery, extent of aortic arch repair, frozen elephant trunk, coronary bypass grafting, number of transfusions, postoperative ventilation, length of ICU stay (intensive care unit), low-cardiac-output-syndrome, postoperative neurologic deficit, delirium, renal replacement therapy. Variables that proved relevant in univariate regression were then included in the multivariate model.

Quality of life was assessed in survivors in long-term follow-up using the SF-36 standardized health survey questionnaire. It uses 36 questions to assess quality of life in eight dimensions: Physical Functioning, Role Physical, Pain, General Health, Vitality, Social Functioning, Role Emotional, Mental Health. In each dimension, a value between 0 (minimum) and 100 (maximum) can be achieved. For better interpretation, the results were then compared to an age-adjusted healthy German reference population using three age groups: <60 years, 60–69 years, >69 years [19].

### 2.3. Operative Procedure

All surgeries were performed according to our institutional standard for the surgery of acute aortic dissection by the attending surgeon, comprising an all-comers approach and prompt surgery. Access was gained under general anaesthesia via median sternotomy. Cannulation for cardiopulmonary bypass was established via femoral, axillary, or direct aortic arterial access. Moderate to deep hypothermia was utilised for organ protection during cardio-circulatory arrest. Exclusion of entry tear, aortic root repair, or replacement where necessary, and varying levels of distal aortic repair, ranging from proximal arch to total arch using the frozen elephant trunk, were carried out as applicable.

### 2.4. Statistical Analysis

Categorical data are reported as numbers and percentages and continuous data as means ± one standard deviation or medians and interquartile ranges, if normal distribution was not met as assessed via the Kolmogorov–Smirnov test. Categorical data were compared with the Fisher’s exact test or chi-square test, normally distributed continuous data with the *t*-test, and, if normal distribution was not met, the Mann–Whitney U test was used. Risk factor analysis was performed with uni- and multivariate logistic Cox regression models. Kaplan–Meier estimates were calculated for survival and reintervention rates, and logrank tests were used for comparison. SF-36 quality of life values were calculated for each quality scale separately, divided into the three age groups <60, 60–69, and >69 years, and compared to the healthy German general population as a reference group [19]. Values of *p* < 0.05 were considered statistically significant. The *p*-values for multi-part variables describe overall effects. All calculations were performed using SPSS version 26.0 (IBM SPSS Statistics for Windows, IBM Corp., Armonk, New York, NY, USA) and R version 4.1.2 (R Foundation for Statistical Computing, Vienna, Austria).

## 3. Results

### 3.1. Demographics

Patient numbers increased significantly during the study period, from 9 patients/year in 2000 to 46 patients/year in 2016 (Pearson’s correlation coefficient r = 0.90, *p* < 0.001). Patients were median 64 [21] years old, 65.9% male, and the median BMI was 26.0 kg/m^2^, with no significant differences between groups. Overall, 73.2% were DeBakey type I dissections, and histopathologic analysis revealed cystic medial degeneration in 15.3%. Clinical presentation was also comparable between groups: overall, 7.3% were resuscitated preoperatively, 22.9% presented in cardiogenic shock, and 27.2% had preoperative neurological deficits. However, we observed a significant increase in calculated perioperative risk using the log. EuroSCORE over time, from 24.9 [32.8] in the early to 38.0 [34.2] in the late period (ANOVA, F = 3.49, eta^2^ = 0.13, *p* < 0.0001). Complete demographic data are displayed in Table 1.

### 3.2. Operative Details

Median operative time decreased over the duration of the study from 315 [121] to 299 [98] minutes (*p* = 0.04), whereas circulatory arrest time increased from 25 [31] to 35 [40] minutes (*p* < 0.0001). Cardiopulmonary bypass (median 193 [78] min., *p* = 0.12) and cross-clamp (median 130 [57] min., *p* = 0.61) times did not change significantly over time.

The preferred arterial cannulation site moved from predominantly femoral (decrease: early 68.5% to late 12.4%) to right axillary (increase: early 27.4% to late 76.2%) during the study period, *p* < 0.001. In addition, intraoperative temperature management changed significantly in favour of warmer temperatures (moderate hypothermia > 28 °C: early 57.3%, late 96.1%, *p* < 0.001) in combination with antegrade cerebral perfusion (early 67.2% vs. late 89.3%, *p* < 0.001) in the late period.

At the level of the aortic root, we observed a significant decrease in the use of mechanical conduits (early 34.4% to late 5.0%), and an increase in biological conduits (25.2% to 30.9%) and valve-sparing root replacements (13.7% to 27.3%), *p* < 0.001. The distal extent of surgery evolved significantly into more extended arch replacements, including frozen elephant trunk implantation (7.6% to 25.9%), *p* < 0.001. Complete operative details are summarized in Table 2.

### 3.3. 30-Day Mortality

**Figure 1:**
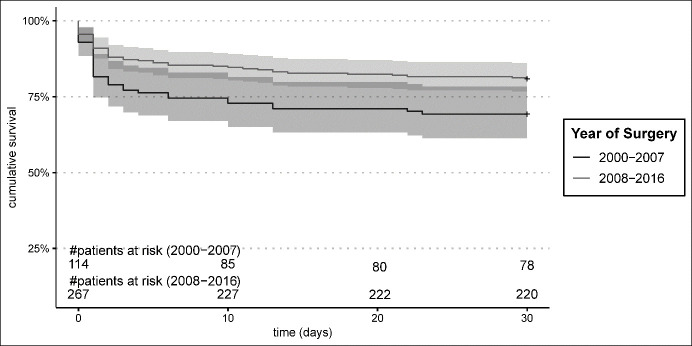
30-day Kaplan–Meier survival estimates.

A significant improvement in 30-day mortality was seen during the study period, from 26.7% in the early group to 17.4% in the late group, *p* = 0.03 (Figure 1). Risk factor analysis revealed BMI (HR 1.06, 95% CI [1.01;1.11], *p* = 0.029), preoperative CPR (HR 7.47, 95% CI [2.65;21.03], *p* < 0.001), postoperative low cardiac output syndrome (HR 9.16, 95% CI [4.70;17.88], *p* < 0.001), and neurological deficit (HR 4.23, 95% CI [1.93;9.27], *p* < 0.001) as predictive in multivariate regression (Table 3). Type or extent of dissection as well as histopathologic evidence of cystic medial degeneration proved to be not predictive for outcomes.

### 3.4. Mortality at Follow-Up

**Figure 2:**
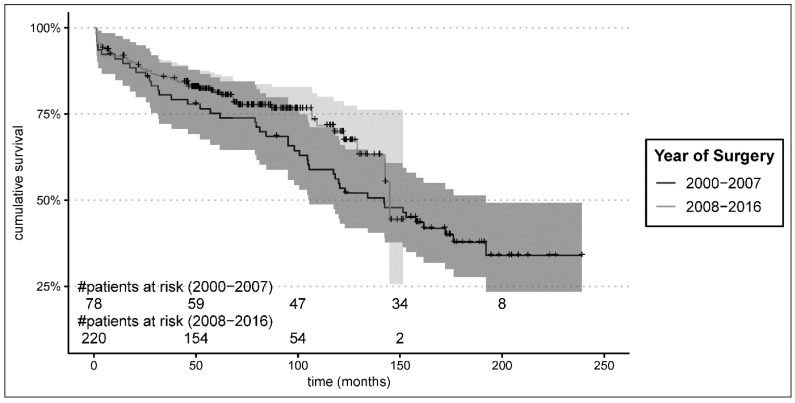
Long-term follow-up Kaplan–Meier survival estimates.

Follow-up was 95.9% complete. Median follow-up time in this analysis was 153.0 [47.4] months. Survival was similar between groups at 1-year follow-up, with 92.3% in the early group and 91.8% in the late group (*p* = 0.91), and did not differ significantly at 5 years (early 75.2% versus late 81.0%, *p* = 0.29), but it proved significantly superior for the late group at 10-year follow-up time: 69.7% compared to 53.4% in the early group, *p* = 0.04 (Figure 2). However, after a complete 12.5 years of follow-up, the survival difference was no more apparent (*p* = 0.15), most likely due to the small numbers (only two patients at risk). The predictive risk factors in multivariate cox regression analysis were age (HR 1.06, 95% CI [1.04; 1.08], *p* < 0.001) and length of ICU stay only (HR 1.04, 95% CI [1.03; 1.05], *p* < 0.001); see Table 4.

### 3.5. Reintervention

**Figure 3:**
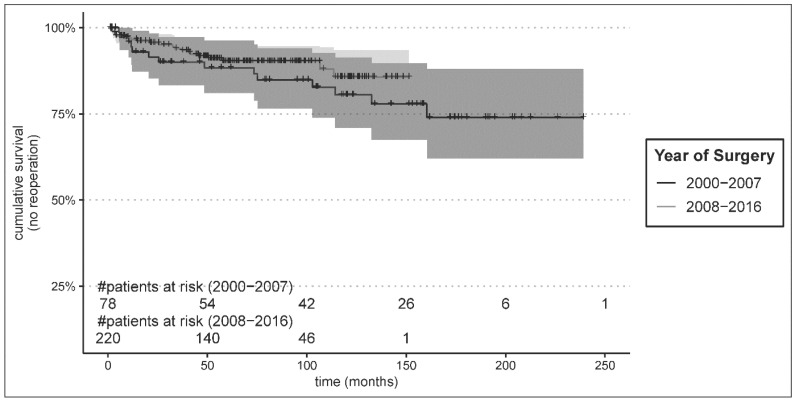
Kaplan–Meier estimates for follow-up freedom from reintervention.

Long-term freedom from aortic reoperation/reintervention was favourable and did not differ significantly between groups: early 74.0% versus late 85.7%, *p* = 0.28 (Figure 3).

### 3.6. Quality of Life

**Figure 4:**
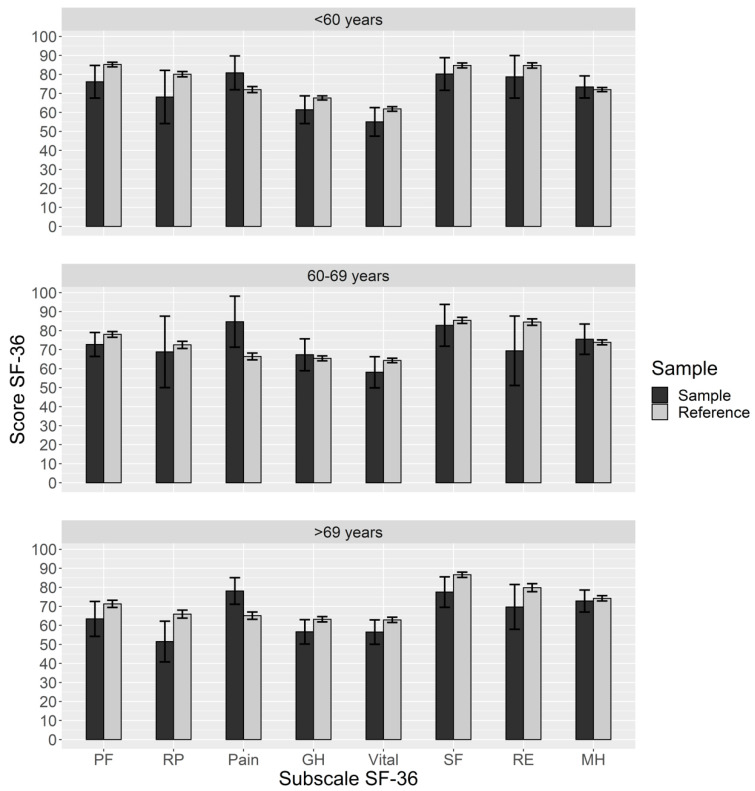
SF-36 quality of life diagram. SF-36, short form 36 health survey questionnaire; PF, physical functioning; RP, physical role; Pain, bodily pain; GH, general health perception; Vital, energy/vitality; SF, social functioning; RE, emotional role; MH, mental health.

Complete SF-36 questionnaires were available for 119 survivors in long-term follow-up. Age-adjusted quality of life according to the SF-36 questionnaire was overall slightly impaired for all but one of the eight dimensions when compared to a healthy general reference population (*n* = 7988). However, statistically significant reduced measures could be found in only two subgroups, ‘pain’ (*p* = 0.02) and ‘emotional role’ (*p* = 0.04), in the age group 60–69 years; see Table 5. The best values for the dissection patients are observed in the ‘mental health’ category, with comparable results to the reference population; see Figure 4.

## 4. Discussion

Over a 17-year observation period and median 12.5-year follow-up time, we identified several important findings:

At our centre, we observed a significant increase of annual type A dissection surgeries over time, with approximately 45 cases per year at present. A similar trend is observed by aortic surgeons worldwide, e.g., reported by Smedberg et al. from Sweden and Abe et al. from Japan [11,20]. However, to date, there is still uncertainty regarding if it is (1) a real increase in aortic dissections, and if so, what are the reasons, (2) an increase in correctly diagnosed aortic dissections sent to surgery in a timely manner, as supported by autopsy data [21,22], or (3) an extension of indication for surgery—30 years ago hardly anyone would have considered performing type A dissection surgery on octogenarians [23]. It is most likely a combination of multiple factors. However, the increasing demand for aortic dissection surgeries necessitates sufficient capacities for a structured, patient-centred health care at dedicated aortic centres [1,24].

Our data document a significant decrease of 30-day mortality despite a significant increase in calculated operative risk using the EuroSCORE. While the 26.7% 30-day mortality rate in the early group corresponds well with the calculated risk of 24.9% using the EuroSCORE, observed mortality in the late group is far below the estimate (observed 17.5% vs. calculated 38.0%). The EuroSCORE is one of the oldest and most widely used calculators to estimate perioperative risk in cardiac surgery. It has been proven that it overestimates actual risks. Even though the more recently introduced GERAADA score might be more accurate in calculating aortic dissection-related perioperative risk, both scores tend to overestimate perioperative risk [7,25]. However, a recent analysis by Ma et al. found a better predictive value for the EuroSCORE II compared to the GERAADA score regarding surgical mortality [26].

Our observed 30-day mortality rates compare well to the “Western world” international data, whereas Asian groups consistently report superior survival rates [1,6-11]. While surgeon- and centre-volume-dependent differences in outcomes are well described, no clear recommendation can be concluded in terms of whether to operate as soon as possible after having confirmed the diagnosis or if a transfer to a specialized aortic centre is justified, weighing the risk of posing the patient at additional risk for a longer transfer [27,28,29]. Moreover, other determinants, like malperfusion of a different extent, have a significant impact on survival probabilities [30].

In our cohort, significant changes in operative strategies were followed over time: For perioperative organ protection, we moved from deep to moderate hypothermia in combination with the consequent standardized application of cerebral perfusion, as supported by contemporary evidence and guidelines [1,31]. In parallel, the predominant arterial cannulation site for cardiopulmonary bypass switched from the femoral to the right axillary artery, facilitating antegrade cerebral perfusion during circulatory arrest. In terms of aortic root management, we saw a decline in mechanical conduits and a rise in valve-sparing root replacements. At the level of the aortic arch, significantly more extensive aortic arch surgery was performed—including frozen elephant trunk implantations. As a consequence, circulatory arrest times prolongated significantly. Interestingly, overall operative times shortened significantly without significant differences in cardiopulmonary bypass or crossclamp times, most likely attributable to modern point-of-care substitution of coagulants. Overall, despite a growing complexity of surgical procedures, mortality rates decreased. However, none of these perioperative modifications proved to be a predictive factor for early or late survival in multivariate regression risk factor analysis. We conclude that it is the synergy of all modifications together rather than one measure alone making the difference [32]. In addition, despite all these improvements in management, the significant and predictive risk factors for survival remain the severity of disease and preexisting conditions the patient presents with at the time of the emergent surgery. Therefore, the GERAADA score could gain more importance when it comes to making the informed decision regarding whether to operate or not [25,33].

Long-term survival at >10 years proved to be superior for the late group compared to the early group (69.7% versus 53.4%). Age and length of ICU stay were the only predictive factors in risk factor analysis. Three contributing factors may play a major role: (1) The greater extent of surgery might improve long-term durability of repair, which is often claimed but has not been proven so far. (2) The establishment of an aortic ambulance warrants a stringent follow-up surveillance for the timely recognition and therapy of late complications. (3) With the advances in endovascular therapy, distal reinterventions only rarely necessitate open surgery, improving the periprocedural safety and survival of patients. Recently published GERAADA data showed similar results, displaying a 10-year-survival rate of 68.3%, which even proved age- and risk-adjusted comparable to the healthy general reference population for low-risk patients according to GERAADA score. Long-term survival differed between GERAADA risk score groups [34]. In contrast, we observed improved long-term survival rates, despite significant higher calculated risk using the EuroSCORE. However, we were not able to find a correlation between evolution in surgical technique and improvement in long-term survival, suggesting a multi-factorial causation.

When we assessed quality of life in the long-term and compared it to the general German reference population, we found it was only slightly impaired in contrast to international data reporting significant limitations in mental health, physical activity, and elevated rates of anxiety and depression. However, existing data mostly relate to shorter follow-up surveillance [19,35,36]. Aortic dissection seems to have a relevant acute impact on patients’ life, emphasizing the need for a better psychosocial or “holistic” approach to postoperative patient care. In the long-term, we could demonstrate that the limitations in quality of life after dissection surgery seem to dissipate over time.

Of note, freedom from reintervention was not significantly different after a median follow-up time of 12.5 years, although a trend might be assumed as Kaplan–Meier reintervention curves can be seen to deviate over time. However, compared to international literature reporting substantial aortic reintervention rates from 19% at one year up to 30–64% within 3–5 years, our overall reintervention rates are remarkably low, with only 15% in >10 years in the contemporary cohort [37,38,39]. Nevertheless, so far, our data do not support the propagated long-term benefits regarding reduction of reintervention rates with more extensive acute surgery, e.g., using the frozen elephant trunk. In conclusion, the benefits of a more extensive operative approach will most likely only be effective for young dissection patients with a long life-expectancy.

## 5. Conclusions

Surgery for type A aortic dissection continues to carry significant morbidity and mortality risks, which are only slowly improving. Long-term follow-up surveillance is key to preventing subsequent complications, whilst quality of life is significantly impaired, even in the long-term, and therefore deserves greater attention.

## 6. Limitations

Due to the retrospective nature of the study, the results may be inadvertently biased. In addition, as a single-centre study, the number of patients is limited, and even the overall 12.5-year follow-up time might not be sufficient to detect all long-term effects of therapy.

## Figures and Tables

**Table 1:** Demographics.

Variable	All Patients (n = 413)	Early Group (n = 131)	Late Group (n = 282)	*p*-Value
Age (years), median [IQR]	64 [21]	63 [22]	65 [20]	0.19
Male sex, % (n)	65.9 (272)	66.4 (87)	65.6 (185)	0.87
BMI (kg/m^2^), median [IQR]	26.0 [5]	26.5 [5]	26.0 [5]	0.34
DeBakey type I, % (n)	73.2 (300)	84.4 (108)	68.1 (192)	0.01
Svensson classification, % (n)				0.01
- class I	78.8 (316)	72.5 (87)	81.5 (229)	
- class II	12.2 (49)	22.5 (27)	7.8 (22)	
- class III	1.5 (6)	0.8 (1)	1.8 (5)	
- class IV	3.7 (15)	1.7 (2)	4.6 (13)	
- class V	3.7 (15)	2.5 (3)	4.3 (12)	
Cystic medial degeneration, % (n)	15.3 (63)	19.1 (25)	13.5 (38)	0.07
Preop. atrial fibrillation, % (n)	7.7 (30)	5.2 (6)	8.7 (24)	0.54
Preop. arterial hypertension, % (n)	88.0 (345)	82.3 (93)	90.3 (252)	0.04
Preop. diabetes, % (n)	8.1 (31)	11.9 (13)	6.6 (18)	0.10
Preop. COPD, % (n)	8.4 (33)	5.1 (6)	9.7 (27)	0.11
Re-operation, % (n)	5.7 (23)	3.2 (4)	6.7 (19)	0.29
Preop. CPR, % (n)	7.3 (29)	7.7 (9)	7.1 (20)	0.84
Preop. CS, % (n)	22.9 (91)	25.9 (30)	21.6 (61)	0.36
Preop. ventilation, % (n)	15.7 (63)	16.7 (20)	15.2 (43)	0.76
Preop. neurologic deficit, % (n)	27.2 (105)	30.1 (34)	26.0 (71)	0.42
Preop. malperfusion, % (n)	30.4 (118)	35.5 (39)	28.4 (79)	0.18
Log. ES (%), median [IQR]	36.3 [35.3]	24.9 [32.8]	38.0 [34.2]	< 0.0001

Continuous variables are given as median [interquartile range] and categorical variables as percentages (count). n, number; BMI, body mass index (kilogram per square meter); Preop., preoperative; COPD, chronic obstructive pulmonary disease; CPR, cardio-pulmonary resuscitation; CS, cardiogenic shock; Log. ES, logistic EuroSCORE (European system for cardiac operative risk evaluation).

**Table 2:** Operative details.

Variable	All Patients (n = 413)	Early Group (n = 131)	Late Group (n = 282)	*p*-Value
Skin-to-skin time (min), median [IQR]	305 [110]	315 [121]	299 [98]	0.04
CPB time (min), median [IQR]	193 [78]	185 [89]	197 [73]	0.12
Crossclamp time (min), median [IQR]	130 [57]	125 [58]	132 [57]	0.61
Circulatory arrest time (min), median [IQR]	35 [35]	25 [31]	35 [40]	<0.0001
Graftdiameter (mm), median [IQR]	28 [3]	28 [4]	28 [2]	0.26
Arterial cannulation site, % (n)				<0.001
- femoral	29.6 (120)	68.5 (85)	12.4 (35)	
- axillary	61.3 (239)	27.4 (34)	76.2 (215)	
- aortic	9.1 (37)	4.0 (5)	11.3 (32)	
Temperature, % (n)				<0.001
- deep hypothermia (<28 °C)	15.4 (63)	42.0 (55)	2.9 (8)	
- moderate hypothermia (>28 °C)	84.7 (347)	57.3 (75)	96.1 (268)	
Antegrade cerebral perfusion, % (n)	82.8 (331)	67.2 (80)	89.3 (251)	<0.001
Aortic root procedure, % (n)				<0.001
- mechanical conduit	14.3 (59)	34.4 (45)	5.0 (14)	
- biological conduit	29.1 (129)	25.2 (33)	30.9 (87)	
- valve-sparing (David)	23.0 (95)	13.7 (18)	27.3 (77)	
Aortic arch procedure, % (n)				<0.001
- hemiarch replacement	28.6 (118)	19.8 (26)	32.6 (92)	
- total arch replacement	34.6 (143)	26.7 (35)	38.3 (108)	
- frozen elephant trunk	20.1 (83)	7.6 (10)	25.9 (73)	

Continuous variables are given as median [interquartile range], categorical variables as percentages (count). n, number; CPB, cardio-pulmonary bypass; °C, degree Celsius.

**Table 3:** Risk factor analysis for 30-day mortality.

Variable	Univariate HR [95%-CI]	*p*-Value	Multivariate HR [95%-CI]	*p*-Value
BMI	1.07 [1.03;1.11]	0.001	1.06 [1.01;1.11]	0.029
Preop. CPR	4.01 [2.29;7.04]	<0.001	7.47 [2.65;21.03]	<0.001
Preop. cardiogenic shock	2.69 [1.72;4.20]	<0.001		
Preop. malperfusion	2.64 [1.66;4.17]	<0.001		
Preop. neurologic deficit	2.39 [1.43;4.00]	0.001		
Mild hypothermic circulatory arrest	0.57 [0.34;0.96]	0.036		
Hemiarch	0.50 [0.26;0.95]	0.035		
Coronary artery bypass grafting	3.59 [2.05;6.30]	<0.001		
Low cardiac output syndrome	7.52 [4.77;11.85]	<0.001	9.16 [4.70;17.88]	<0.001
Postop. neurological deficit	2.69 [1.55;4.68]	<0.001	4.23 [1.93;9.27]	<0.001

HR, hazard ratio; CI, confidence interval; BMI, body mass index; Preop., preoperative; CPR, cardio-pulmonary resuscitation; Postop., postoperative.

**Table 4:** Risk factor analysis for mortality in long-term follow-up.

Variable	Univariate HR [95%-CI]	*p*-Value	Multivariate HR [95%-CI]	*p*-Value
Age	1.06 [1.04;1.08]	<0.001	1.06 [1.04;1.08]	< 0.001
Diabetes	2.72 [1.25;5.89]	0.011		
Chronic obstructive pulmonary disease	2.15 [1.03;4.46]	0.040		
Valve-sparing root replacement	0.48 [0.26;0.90]	0.021		
Transfusions	1.03 [1.02;1.05]	<0.001		
Postop. ventilation > 24 h	2.00 [1.08;3.70]	0.028		
ICU stay	1.03 [1.02;1.04]	<0.001	1.04 [1.03;1.05]	<0.001
Low cardiac output syndrome	2.80 [1.71;4.44]	<0.001		
Postop. renal replacement therapy	3.18 [2.10;4.83]	<0.001		
Postop. neurological deficit	3.04 [1.81;5.10]	<0.001		

HR, hazard ratio; CI, confidence interval; Postop., postoperative; h, hours; ICU, intensive care unit.

**Table 5:** SF-36 quality of life table.

SF-36 Dimension	Age Group	Study Group (n = 119)	Reference Population (n = 7988)	*p*-Value
Physical Functioning	<60 years	76.1 [67.5; 85.9]	85.2 [84.0; 86.4]	0.07
	60–69 years	72.7 [66.4; 83.1]	78.0 [76.5; 79.5]	0.36
	>69 years	63.4 [54.2; 73.4]	71.3 [69.4; 73.3]	0.33
Physical Role	<60 years	68.1 [54.1; 80.3]	80.1 [78.7; 81.6]	0.05
	60–69 years	68.8 [50.0; 84.6]	72.5 [70.6; 74.4]	0.64
	>69 years	51.5 [40.8; 63.5]	65.9 [63.8; 68.1]	0.11
Pain	<60 years	80.8 [71.9; 88.8]	72.0 [70.4; 73.6]	0.19
	60–69 years	84.7 [71.3; 95.3]	66.4 [64.6; 68.2]	0.02
	>69 years	78.1 [71.1; 84.7]	65.1 [63.2; 67.1]	0.11
General Health	<60 years	61.4 [54.1; 68.9]	67.6 [66.5; 68.7]	0.18
	60–69 years	67.3 [58.9; 75.5]	65.4 [64.1; 66.8]	0.74
	>69 years	56.6 [50.2; 63.0]	63.2 [61.8; 64.5]	0.24
Vitality	<60 years	55.0 [47.5; 62.2]	61.8 [60.6; 62.9]	0.16
	60–69 years	58.1 [49.9; 66.0]	64.3 [63.1; 65.4]	0.20
	>69 years	56.5 [50.1; 63.4]	62.9 [61.5; 64.4]	0.29
Social Functioning	<60 years	80.2 [71.6; 87.5]	84.7 [83.4; 86.0]	0.41
	60–69 years	82.8 [71.8; 92.0]	85.4 [83.8; 86.9]	0.72
	>69 years	77.5 [69.5; 85.5]	86.6 [85.2; 87.9]	0.11
Emotional Role	<60 years	78.7 [67.5; 88.3]	84.7 [83.3; 86.4]	0.36
	60–69 years	69.4 [51.1; 85.7]	84.5 [82.8; 86.2]	0.04
	>69 years	69.7 [57.9; 80.9]	79.8 [77.7; 81.9]	0.25
Mental Health	<60 years	73.4 [67.6; 78.8]	72.0 [70.9; 73.1]	0.76
	60–69 years	75.5 [67.5; 82.1]	73.8 [72.5; 75.0]	0.75
	>69 years	72.8 [67.0; 78.8]	74.2 [72.8; 75.5]	0.81

SF-36, short form 36 health survey questionnaire. Values of SF-36 survey are given as means [95%-confidence interval] for each of the eight dimensions and three age groups.

## Data Availability

Due to legal reasons, the data presented in this study are only available upon reasonable request from the corresponding author.
